# A Pipeline for the Isolation and Cultivation of Microalgae and Cyanobacteria from Hypersaline Environments

**DOI:** 10.3390/microorganisms13030603

**Published:** 2025-03-05

**Authors:** Petra Tavčar Verdev, Marko Dolinar

**Affiliations:** Department of Chemistry and Biochemistry, Faculty of Chemistry and Chemical Technology, University of Ljubljana, 1000 Ljubljana, Slovenia

**Keywords:** salterns, salt pans, halophilic microorganisms, culture systems, axenic culture, biotechnology, Chlorophyta, Cyanoprokaryota

## Abstract

Microorganisms in high-salinity environments play a critical role in biogeochemical cycles, primary production, and the biotechnological exploitation of extremozymes and bioactive compounds. The main challenges in current research include isolating and cultivating these microorganisms under laboratory conditions and understanding their complex adaptive mechanisms to high salinity. Currently, universally recognized protocols for isolating microalgae and cyanobacteria from salt pans, salterns, and similar natural habitats are lacking. Establishing axenic laboratory cultures is essential for identifying new species thriving in high-salinity environments and for exploring the synthesis of high-value metabolites by these microorganisms ex situ. Our ongoing research primarily focuses on photosynthetic microorganisms with significant biotechnological potential, particularly for skincare applications. By integrating data from the existing literature with our empirical findings, we propose a standardized pipeline for the isolation and laboratory cultivation of microalgae and cyanobacteria originating from aqueous environments characterized by elevated salt concentrations, such as solar salterns. This approach will be particularly useful for researchers working with microorganisms adapted to hypersaline waters.

## 1. Introduction

Although extreme salt concentrations are generally believed to inhibit microbial growth, natural environments with elevated salt levels are rich in microorganisms that have adapted to these specific conditions. Among these halophiles are archaea, bacteria (including cyanobacteria), and eukaryotic organisms, such as green microalgae.

Microalgae and cyanobacteria are important sources of high-value metabolites, including carotenoids, polyunsaturated fatty acids (e.g., eicosapentaenoic acid (EPA) and docosahexaenoic acid (DHA)), phytosterols, phycocolloids, sulfated polysaccharides, antioxidants, proteins (such as phycobiliproteins), peptides, mycosporine-like amino acids, polyphenols, glycerol, and more [1,2,3,4,5,6,7]. Due to the high diversity of marine microalgae and their global distribution, the so-called ‘blue biotechnology’ has become increasingly popular among researchers and industry partners. The main idea is to exploit the benefits microalgae offer in fields such as pharmaceuticals, food supplements, cosmetics, biofuel production, bioremediation, and wastewater treatment [1,8,9,10,11,12].

Microalgae and cyanobacteria have several desirable characteristics for biotechnological applications [4,13,14,15]:

I. They are photosynthetic organisms with short generation times, allowing for rapid and environmentally friendly biomass production;
II. They are highly adaptable to changing conditions and survive in unfavorable environments, which is particularly true for extremophiles, including species living in hypersaline environments;
III. They exhibit high metabolic plasticity, meaning they can adjust their biochemical profile in response to different abiotic factors (e.g., nutrient depletion, increased light intensity, or higher salinity). This makes it easy to manipulate the production of desired secondary metabolites;
IV. Microalgae cultivation in open ponds, photobioreactors, or biofilm systems is relatively inexpensive and easy to manage.

These characteristics make large-scale microalgae cultivation generally more cost effective than plant biotechnology and more sustainable than extracting bioactive compounds from animal sources. The commercial success of microalgae biotechnology relies heavily on identifying species that produce desired metabolites and understanding their physiology [16].

Metagenomic analysis can provide insights into the microbial diversity of different environments and the abundance of specific species within those environments [17]. Diversity studies demonstrate very high variations between salterns and salt lakes with different salt concentrations. Most of the work was performed on metabarcoding of prokaryotes and these analyses revealed that in some hypersaline environments, archaea significantly prevail over bacteria while in some others, the opposite was observed. For illustration, a recent study of microorganisms in artificial salterns in Vietnam and their comparison to Italian saltern samples has shown that in 19 analyzed hypersaline environments, the number of amplicon sequence variants for 16S rDNA metabarcoding was between 59 and 542, the average being 256. Even in crystallized salt, the diversity was high, ranging from 59 to 358 amplicon sequence variants [18]. Despite this diversity, commercial exploitation is often restricted to microorganisms that can adapt well to laboratory conditions and are good candidates for cultivation in industrial settings.

The main goal of laboratory cultivation is to obtain axenic cultures, i.e., pure cultures of a single strain or species without any contaminating organisms. These cultures enable precise investigation of their morphology, physiology, adaptation mechanisms, taxonomy, biochemical properties, and biotechnological potential. Although many hypersaline microalgae species exist, only a small fraction have been successfully established as axenic cultures and are available in culture collections. Additionally, it is estimated that many metabolites from extremophile microalgae have yet to be discovered [4].

Despite numerous efforts to isolate pure microbial cultures from high-salinity environments, no standardized isolation procedure currently exists. In this article, we thus present guidelines specifically designed for the isolation and laboratory cultivation of photosynthetic microorganisms from hypersaline environments, with a focus on biotechnologically relevant green microalgae and cyanobacteria (historically known as blue-green algae).

## 2. Hypersaline Environments as Highly Dynamic Habitats for Microorganisms

Salterns and other hypersaline ecosystems, such as salt pans, salt flats, salt lakes, and saline rivers, are globally distributed habitats that harbor a remarkable diversity of microorganisms adapted to high salinity conditions. Salterns are man-made facilities designed for salt production from seawater or brine. Typically, they are constructed as a series of interconnected shallow ponds where seawater is allowed to evaporate naturally, resulting in salt crystallization. Due to their coastal locations, salterns form unique environmental conditions at the intersection of marine and terrestrial habitats [19].

One key characteristic of these environments is their constantly changing nature. Salinity levels vary widely throughout the year due to water evaporation and the inflow of rainwater and seawater, often changing rapidly in short periods. This variability creates niches for diverse microorganisms with different salt tolerances to thrive. When salinity is low to moderate, many halotolerant species are present. However, when salinity becomes extremely high, typically in the summer, halophiles dominate these environments. Halophilic microorganisms exhibit a wide range of metabolic abilities to adapt to these particular conditions [20,21,22]. In addition to salinity, factors like UV irradiation, water temperature, pH, low rate of oxygen diffusion, and limited nutrient availability also affect microbial growth and diversity in hypersaline ecosystems [10]. Furthermore, water levels in crystallization ponds are typically low and decrease further during warm weather, sometimes leaving the pond bottoms nearly dry. Several environmental factors (high salinity, high temperature, low oxygen availability) act synergistically when water evaporates in salterns or salt ponds. Effects can be seen at the level of cell structure adaptations and changes in metabolic processes [23], as well as in the microbial community composition [24]. Systematic studies focused on synergistic effects on microalgae and cyanobacteria are still missing though.

Microbial adaptations to extreme conditions have a significant biotechnological potential, as previously shown e.g., for microbes from the hypersaline environments in the Alicante region in Spain [10].

Another peculiarity of salt pans and of some salterns (such as the Sečovlje salterns in the Northern Adriatic, which we focused on) is the presence of a specialized microbial mat, a multilayered ecosystem composed of a diverse community of microorganisms. Biodiversity studies have shown that these self-sustaining habitats are populated by a wide variety of microorganisms, such as archaea, bacteria (including cyanobacteria), and microalgae [25,26,27,28]. The hypersaline benthic microbial mat, known as the ‘petola’, is found at the bottom of crystallization ponds in some of the Mediterranean solar salterns. The petola mat plays an important role in solar salt production and has been traditionally cultivated since the 14th century. It helps protect and stabilize the surface of crystallization ponds and is believed to enhance the whiteness of the salt, resulting in higher-quality salt production [25].

In the Sečovlje salterns, the northernmost operating salterns of the Adriatic Sea, changes in the biological composition of the petola are closely linked to the salt production cycle. The elemental and microbial composition of the petola varies with the season and is influenced by the application of a layer of fresh marine mud, traditionally completed in the spring to replenish nutrients for the microorganisms in the petola [25]. Other than microbial mats, other complex substrates, such as rocks, clay, and sediments, can be found in hypersaline environments.

## 3. Microalgae and Cyanobacteria in High-Salinity Environments

Over the past two decades, the biodiversity of microorganisms in various hypersaline environments has been substantially studied [17,26,27,29,30,31]. An important goal of this research is to identify and utilize extremophilic bacteria, archaea, and microalgae for biotechnological applications. Generally, increased salinity correlates with reduced biodiversity as only a few taxa are well-adapted to extreme salt stress and display unique genotypic and phenotypic characteristics [32,33,34,35,36,37].

Halophilic microorganisms have evolved various strategies to cope with extreme conditions, including the production of compatible solutes to maintain cellular osmotic balance, increased expression or reorganization of specialized transport systems for salt ions, mechanisms to limit UV-induced damage, and the increased synthesis of secondary metabolites (such as antioxidants and polysaccharides) that facilitate microbial survival and growth in harsh conditions [20,22,38,39]. Namely, green microalgae from the genus *Dunaliella*, which commonly inhabit hypersaline environments, dynamically regulate intracellular glycerol concentration to control internal osmolarity and prevent cell death under both hyper- and hypo-osmotic stress [40]. Additionally, *Dunaliella* species lack a rigid cell wall, allowing them greater morphological adaptability to environmental stress [41], and exhibit many adaptations at the genomic level [42]. Furthermore, marine species from different kingdoms produce mycosporine-like amino acids, which act as UV-absorbing compounds to protect against high levels of UV radiation [43]. Notably, mycosporine-like amino acids are becoming increasingly popular in skincare applications due to their antioxidant and photoprotective properties [44].

The most abundant photosynthetic organisms found in saline environments are members of the Chlorophyta (green algae) and Cyanoprokaryota (cyanobacteria) groups [33,35,45]. The most prominent eukaryotic inhabitants of hypersaline ecosystems are the unicellular green algae of the genus *Dunaliella.* Many species of *Dunaliella*, such as *D. salina*, *D. viridis*, *D. parva*, *D. bioculata*, *D. percei*, and others, are well-known for their halophilic properties [41,46,47]. *Dunaliella salina* is already widely used in biotechnology for the production of β-carotene. However, other species of *Dunaliella* can also accumulate various carotenoids, including β-carotene, α-carotene, lutein, and zeaxanthin, under specific growth conditions, such as high light intensity, high concentrations of NaCl, or nitrate deficiency [48,49,50]. In addition to *Dunaliella*, several other photosynthetic microorganisms present in hypersaline environments have been explored for their biotechnological potential (summarized in Table 1). Many unicellular and non-heterocystous filamentous cyanobacteria have been identified and isolated from hypersaline environments, where they typically form multi-layered benthic microbial mats [34]. Glavaš and colleagues conducted a 16S rRNA gene sequence analysis, revealing that cyanobacteria are the predominant bacterial species in the petola mat of the Sečovlje salterns, with the composition of cyanobacterial communities varying slightly between low- and high-salinity seasons [25]. Additionally, a study by Meier and co-workers found different filamentous cyanobacteria form salt-crust-covered microbial mats along the coast of Oman [26]. Most halophilic cyanobacterial species belong to the genera *Coleofasciculus*, *Phormidium*, *Geitlerinema*, *Lyngbya*, *Oscillatoria*, *Spirulina*/*Halospirulina*, *Nostoc*, and *Synechococcus* [25,26,27,34,51]. It was shown that cyanobacteria generally prefer moderate salt concentrations, and their growth is inhibited at extremely high salinity (salt concentrations of approximately 20% and above). A study of the solar saltern in Sfax (Tunisia) showed that cyanobacterial abundance significantly decreased compared to green algae at very high salinities, where they constituted only 0.5% of the total photosynthetic microalgae [36]. Similarly, in hypersaline microbial mats from the coastal region of Oman, cyanobacterial abundance and photosynthetic activity were greatly reduced under salt-saturation conditions [26]. Extreme salinity levels inhibit oxygenic photosynthesis, favoring anoxygenic phototrophs [26]. Interestingly, the common hypersaline mat-forming cyanobacterium *Coleofasciculus chthonoplastes* (formerly *Microcoleus chtonoplastes*) can shift to phototrophic sulfide oxidation (anoxygenic photosynthesis) and exhibits a high tolerance to sulfide, which is toxic to most cyanobacteria as it inhibits oxygenic photosynthesis [52,53].

In contrast to *Dunaliella*, hypersaline cyanobacterial strains are not used regularly for biotechnological applications. This is partly due to their complex taxonomy, which makes precise species identification challenging and limits our understanding of their biochemical properties [54]. Additionally, many cyanobacteria are known to produce toxins harmful to humans and animals [55], making them less suitable for industrial use compared to eukaryotic photosynthetic microorganisms due to associated health risks. Therefore, further basic research is needed to identify and characterize the most promising hypersaline cyanobacterial species and strains for the production of high-value compounds.

**Table 1:** Currently exploited and proposed halophilic cyanobacteria and green algae for biotechnological production.

Microalgae	Important Metabolites	Biotechnological Applications	References
*Dunaliella*	β-carotene and other carotenoids, glycerol, lipids and fatty acids, antimicrobial compounds	Food industry, cosmetics, pharmaceuticals, biofuel production	[46,48,49,50]
*Asteromonas*	Lipids	Biodiesel production	[56]
*Nannochloropsis*	Lipids, polyunsaturated fatty acids, pigments	Biofuel production, animal feed	[57,58,59]
Cyanobacteria	Mycosporine-like amino acids, antimicrobial and antiviral compounds, pigments	Cosmetics, nutraceuticals, food industry	[5,6,14,44]

## 4. Current Methods for the Isolation and Cultivation of Halophilic Microalgae: Key Challenges

While metagenomic analysis provides a comprehensive approach to revealing the entire microbial community at a particular location, cultivating these microorganisms under artificial conditions often remains a bottleneck in harnessing their bioactive potential. Establishing axenic (pure) cultures of a specific species or strain is crucial for its identification and taxonomic classification, as well as for studying its morphology, physiology, and production of high-value metabolites. Moreover, axenic cultures are essential for determining whether a particular microalgal species is suitable for industrial applications. For most biotechnological purposes, it is important that a specific microalga, which produces a molecule of interest, can grow efficiently—rapidly and with minimal effort—in open ponds or other large-scale growth settings [15]. Preparing axenic cultures in the laboratory is thus the first step toward species characterization and subsequent biotechnological utilization.

### 4.1. Sampling in the Hypersaline Environments

Sampling microalgae and cyanobacteria in salt pans is similar to sampling photosynthetic microorganisms in other marine or freshwater habitats. However, several specific considerations must be considered when sampling in solar salterns or other hypersaline environments:

I. Water level in the sampling area: The water level in crystallization pools is often low or even absent, particularly during the summer months. In addition, halite crystals formed during brine crystallization can entrap surrounding microorganisms, providing unique opportunities for alternative sampling strategies;
II. Salinity variations: The salinity of the water fluctuates significantly throughout the year and should be measured during sampling to ensure the right conditions for laboratory cultivation;
III. Soil sediments: Collected samples of the microbial mat often contain mud and/or sediment (lower part of the samples), resembling wet soil samples. The presence of sediments and related impurities affects subsequent steps of the isolation process;
IV. Diversity of sample types: The isolation of photosynthetic microorganisms from water samples and benthic microbial mats typically requires different approaches to effectively extract distinct subsets of microorganisms from these samples. Additionally, laboratory growth conditions must be adjusted based on factors such as the season of sample collection and other environmental variables.

Brine is sampled using either sterile syringes (for volumes up to 50 mL) or thoroughly washed and sterilized bottles (250 mL to 1000 mL). The equipment for collecting microbial mats varies depending on the surface being studied. Soft or muddy surfaces are sampled using core samplers (corers) with a diameter of 4–8 cm. For mats on solid surfaces (e.g., rocks), spatulas or scoops are used. The collected mat is then transferred to a sterile container of appropriate size or disposable sealable plastic bags. Crystallized salt is sampled with a clean scoop. During sampling, gloves are worn and all sampling parameters (such as temperature, pH, salinity, time, and exact location) are recorded. Samples are transferred to the laboratory as quickly as possible to avoid overheating or spoilage.

Typically, crude environmental samples are first dissected (if needed) using a scalpel, forceps, or needles and then partially homogenized using methods such as grinding, vortexing, or homogenization with glass beads to break up microalgal clumps and/or cyanobacterial filaments. This is often followed by filtering through membranes with known pore sizes or by density gradient centrifugation to achieve the partial separation of specimens or to remove non-specific environmental material from the sample. The resulting suspension is usually enriched in a liquid nutrient medium for 1–2 weeks before being plated on a solid nutrient medium or further purified with other approaches [60,61,62].

Various microbiological techniques are routinely used to isolate specific microalgae from complex environmental samples and obtain axenic cultures. Some of them are micromanipulation and single-cell isolation, streak plating, spread plating, serial dilution, and, less commonly, the pour plate method and fluorescence-activated cell sorting [60,62,63,64]. However, there is no standardized procedure as the optimal approach depends on the specific characteristics of the sample. Importantly, choosing one technique over others can result in the loss of certain strains present in the original sample. For instance, repeated subculturing in a liquid medium through serial dilution typically enriches the predominant species, whereas rarer species are often lost. Therefore, ideally, a combination of techniques should be applied to achieve the best results along with constant monitoring of the culture composition and purity. Nonetheless, obtaining axenic cultures remains a labor-intensive and time-consuming process and is not always successful [61].

### 4.2. Laboratory Settings for the Cultivation of Halophilic Microalgae

For the successful laboratory cultivation of halophilic microalgae and cyanobacteria, suitable growth conditions must be provided. The main parameters to consider include the composition of the nutrient medium, temperature, and illumination settings.

#### 4.2.1. Growth Medium

The choice of growth medium is of paramount importance for cultivation as it defines the range of available nutrients and their abundance, salinity, and pH. A general overview of the available media is beyond the scope of this report. By way of illustration, a recent article [65] on the cultivation of microorganisms from a hypersaline lake lists several usable media and a comprehensive review of media for the growth of halophilic and halotolerant prokaryotes [66] additionally describes the role of different media components. Media for photoautotrophic microorganisms typically contain salts, sources of nitrogen and phosphorous, and standardized trace elements. Boron, an essential microelement for the formation of cyanobacterial heterocysts, is included in the majority of cyanobacterial media in the form of boric acid [61,67]. Conversely, certain metal ions included in trace element solutions, such as copper, can inhibit the growth of specific cyanobacterial strains and may need to be substituted [62]. Liquid media are often supplemented with vitamins, such as thiamine (B1), biotin (B7), and cobalamin (B12), to enhance biomass production [68].

When isolating cyanobacteria and microalgae from muddy environmental samples, such as microbial mats, a small amount of sediment from the sampling site can be added to the selected liquid medium. However, this approach often results in mixed cultures as sediments typically contain diverse microbial communities, necessitating additional purification steps. Alternatively, specialized media containing artificial soil substrates may be used. It is important to note that the composition of micro- and macro-elements in different soils varies widely and commercial soil substrates may not meet the requirements of the isolated specimens [61].

Another important factor affecting the cultivation of halophilic microorganisms is the concentration of salts in the growth medium. Salinity is typically adjusted to the desired level by varying the concentration of NaCl, allowing for the simulation of conditions naturally present in solar salterns, where salinity can fluctuate greatly throughout the year (from about 3.5% salinity of seawater to saturated brines of sodium and calcium chloride) [69]. Sometimes, media supplemented with sterilized seawater can be used as a cost-effective alternative to artificial seawater media [32,36].

Different halophilic species show preferences for different salinities. For instance, *Dunaliella salina* exhibits optimal growth at NaCl concentrations between 1.5 and 3 M while *Dunaliella viridis* and *Dunaliella parva* generally thrive at lower NaCl concentrations of around 1 M [47,70,71]. Since species identification for the genus *Dunaliella* is quite challenging based on microscopic observations and multiple species can coexist in the same hypersaline environment, we recommend using a growth medium with intermediate salinity when handling environmental samples. The salinity should be gradually increased while continuously monitoring algal growth. Furthermore, hypersaline cyanobacteria generally prefer moderate salinities and may enter a dormant state if the salinity of the medium is too high [26].

**Figure 1:**
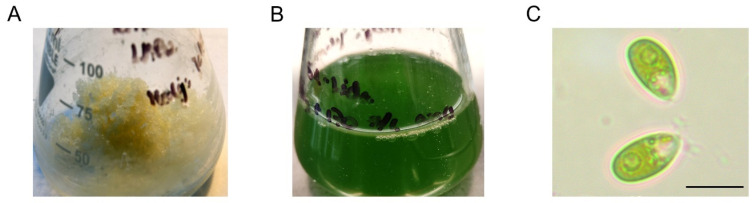
Revival of a dry culture sample of green microalgae *Dunaliella* sp. in our laboratory. (**A**) Initial state of the sample, which was dried up by slow evaporation at room temperature. The culture was maintained in modified Johnson’s medium, supplemented with 170 g/L NaCl, prior to that. (**B**) The same sample one month after adding distilled water to the dried algae. (**C**) Microscopic view of the revived *Dunaliella* sp., showing two ovoid-shaped cells in the vegetative motile form. Scale bar corresponds to 10 µm. Microscopy was performed using a Primostar optical microscope with a 100× Pan-Achromat immersion objective (Zeiss, Jena, Germany).

Interestingly, our experience shows that *Dunaliella* species can also be cultivated from crystallized salt samples. We dried a culture of *Dunaliella* sp. by allowing the nutrient medium to slowly evaporate, leaving the microalgae to dry out, and stored it in this state for approximately 2 years at room temperature under a constant cool white illumination of about 15 μmol photons m^−2^ s^−1^. We then tested whether the dried culture could be revived by adding sterilized distilled water. After approximately two weeks of cultivation at 23 ± 2 °C under constant illumination with cool white light at an intensity of 25 μmol photons m^−2^ s^−1^, the culture turned green, indicating active algal growth. Optical microscopy confirmed the presence of motile, ovoid-shaped cells with two flagella (Figure 1). This is similar to a report of a microalgae isolation attempt conducted by scraping algae from salt crystals collected in India [32]. *Dunaliella* species from salt pans are known for their high tolerance to salt stress and can survive in conditions of up to salt saturation (around 5.5 M NaCl or 35% salinity) [48,72]. Because few halophilic organisms can withstand salt crystallization, isolating *Dunaliella* from salt crystals could potentially allow for a quick establishment of axenic cultures.

Most cyanobacteria and green microalgae are alkaliphiles or neutrophiles [63]. To maintain an appropriate pH in the nutrient medium, buffering agents, such as HEPES (2-[4-(2-hydroxyethyl)piperazin-1-yl]ethane-1-sulfonic acid), can be added [63]. Strains of *Spirulina* (*Arthrospira*), which are commonly used as food supplements, are typically cultivated in alkaline media, such as AO medium with a pH of 9.4 [73] or Zarrouk’s medium with a pH of 9 [74], to achieve the best algal growth. As with other growth parameters, the pH of the medium can significantly affect cell metabolism. For example, when *Spirulina* is grown in a medium with a lower pH (8.5), it produces more carotenoids and chlorophyll a, while a higher pH (9.5) enhances the synthesis of phenolic compounds [74].

The composition of the solid nutrient medium also needs to be considered. Agar can contain substances that inhibit the growth of certain microalgae, particularly cyanobacteria [75]. If cyanobacteria are identified in an environmental sample (e.g., by optical microscopy), it is advisable to cultivate sensitive specimens on agarose plates rather than standard agar plates. Additionally, using a low concentration of agar in combination with thiosulphate when preparing solid media can increase plating efficiency for many cyanobacterial strains [76]. Alternatively, agar can be purified by washing it repeatedly with distilled water and 95% ethanol [62].

The literature describes numerous media formulations to meet the specific needs of different microalgae subsets. For the growth of marine microalgae, ASN-III medium is typically recommended [63], although other media such as MN, ASP-2, BG-11, and Zarrouk’s medium have also been used [77,78,79]. Algae from the genus *Dunaliella* are usually cultivated in Johnson’s medium and its modifications, Conway medium, or F/2 medium [10,80]. It is important to note that these media were originally developed for marine species and should be modified to meet the specific requirements of halophilic microorganisms.

#### 4.2.2. Temperature

Marine green microalgae and cyanobacteria are generally cultivated at temperatures ranging from 20 °C to 25 °C [60,62]. However, man-made solar salterns, which typically have shallow water (usually up to 15 cm), can heat up to higher temperatures, especially during late spring and summer. Optimal growth for *Dunaliella* species has been reported between 25 °C and 35 °C. While most strains can survive temperatures around 40 °C, their growth is typically inhibited under these conditions [48]. Therefore, it is advisable to cultivate crude environmental samples at different temperatures to promote the faster growth of specific subsets of microorganisms under each condition.

#### 4.2.3. Illumination

Illumination settings often vary for different microalgae strains cultivated under laboratory conditions. Generally, marine algae exhibit better growth at lower light intensities (20–30 μmol photons m^−2^ s^−1^) compared to freshwater algae. However, reports on the optimal illumination conditions for specific strains can differ significantly between research groups. Although light intensities in salterns can often be very high, some studies suggest that *Dunaliella salina* should be grown in the laboratory under low light conditions of approximately 30 μmol photons m^−2^ s^−1^ [36] while others recommend higher light intensities, up to 150 μmol photons m^−2^ s^−1^ [10].

It is important to note that high light intensities can trigger a stress response in some strains, leading to changes in their metabolic profiles. Under such conditions, many *Dunaliella* species begin accumulating carotenoids [51,81,82]. A similar light-induced metabolic response has also been observed in halophilic cyanobacteria from the genus *Phormidium* [51].

For cyanobacteria, illumination ranges of 10–75 μmol photons m^−2^ s^−1^, with various light/dark cycles (such as 12:12, 14:10, 16:8, or constant illumination), are commonly reported [62,83]. Typically, laboratory setups use fluorescent lamps to provide cool or warm white light. Interestingly, cyanobacteria use blue light less efficiently compared to eukaryotic photosynthetic organisms [84]. Accumulating evidence suggests that cyanobacterial growth improves under red light [84,85,86]. Therefore, using filters to define the illumination wavelength range may enhance the growth of certain cyanobacterial strains in the laboratory.

#### 4.2.4. Other Factors

Microalgae cultivation in laboratory settings differs significantly from biomass cultivation intended for industrial use. However, when cultivating larger volumes of culture, it is essential to implement systems that allow for constant stirring or agitation, gas exchange, and continuous nutrient supply. Often, photobioreactors represent the junction between laboratory and biotechnological applications (reviewed in [87]).

Determining the appropriate handling approach for environmental samples in the early stages of isolation is crucial. Aquatic samples and microbial mat samples from the bottom of crystallization pools require different treatment methods. Despite these significant differences, such samples are often cultivated in the same way, using liquid nutrient media. However, this approach favors the growth of planktonic microalgae while species that require attachment may be lost. Conversely, microalgae plated on solid nutrient media, especially those with high salt concentrations, eventually dry out and need to be regularly transferred to fresh nutrient plates.

**Figure 2:**
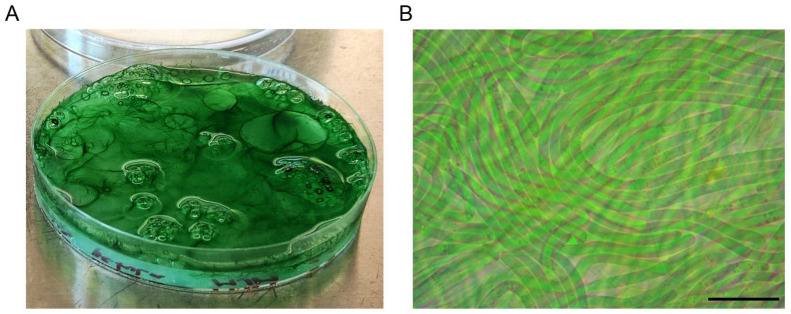
Macroscopic and microscopic view of filamentous cyanobacterium, isolated from the Sečovlje salterns. (**A**) Macroscopic view showing multilayered sheet organization of filamentous cyanobacteria cultivated on a non-nutrient agarose plate topped with liquid nutrient medium. (**B**) Microscopic view of filament organization within the sheet. Scale bar corresponds to 20 µm. Microscopy was performed using a Primostar optical microscope with a 100× Pan-Achromat immersion objective (Zeiss, Germany).

In our opinion, the growth of halophilic filamentous cyanobacteria can be optimized by cultivating them on porous surfaces or materials that resemble their natural habitat. In our lab, we successfully isolated and cultivated a filamentous cyanobacterium from the petola microbial mat (sampled in the Sečovlje salterns, Slovenia) by spreading it on an agarose plate without added nutrients and topping it with a liquid nutrient medium (Figure 2). Fresh medium was added as needed (approximately once a week) to prevent the culture from drying out and the culture was refreshed monthly by transferring a small amount of the cyanobacterial layer to fresh agarose plates. The cyanobacterium eventually covered the entire plate, forming multilayered sheets. The ability to form biofilms and densely packed mats in their natural habitats is a characteristic of many filamentous cyanobacteria [88,89]. Our setup, which mimicked the conditions on the floors of crystallization pools, proved to be effective for the long-term cultivation of isolated filamentous cyanobacterium and we intend to apply this strategy to the laboratory cultivation of other filamentous cyanobacteria as well.

### 4.3. Purification Strategies

Microalgae often coexist and interact with various microorganisms in their microenvironment, including heterotrophic bacteria, fungi, other microalgae, and protozoa [90,91,92]. This diversity can pose challenges when the goal is to obtain an axenic culture containing only a single species or strain. To address this, different purification methods have been developed and continue to be refined (Table 2).

For unicellular planktonic microalgae, the streak plate method is typically effective. This involves multiple instances of re-streaking on fresh solid plates, followed by single colony transfer and maintenance in a liquid medium. Alternatively, serial dilution in a liquid medium can be used for most planktonic species, although this technique often requires a significant amount of time to achieve axenicity [62]. Filamentous cyanobacteria cultures are often mechanically purified by sequentially transferring single filaments from one agar plate to another. To obtain single trichomes, filament-forming cyanobacteria are typically fragmented by vortexing or sonication [62,63,93,94]. Additionally, positive phototaxis (movements towards the light source) can be utilized to separate filamentous cyanobacteria and green algae from accompanying heterotrophs, as the latter cannot move toward light, while cyanobacterial cells can glide toward the illuminated area of a Petri dish [63,78,95].

Furthermore, differences in salinity tolerance among species inhabiting hypersaline environments can be exploited as a purification strategy. For example, *Dunaliella* species, which can tolerate saturation-level salinity, can be selectively enriched compared to mat-forming filamentous cyanobacteria by increasing the salinity of the nutrient medium. It has been demonstrated that saturation-level salinity (approximately 40%) strongly inhibits oxygenic photosynthesis in hypersaline microbial mats [26].

Many filamentous cyanobacteria produce extracellular mucilaginous or gelatinous sheaths that are colonized by heterotrophic bacteria and fungi [96,97,98,99]. While physical methods can accelerate culture purification, they are often insufficient to eliminate persistent contaminants. To purify these cultures, a combination of antibiotics—and if necessary, antimycotics—can be used. Commonly used antibiotics include kanamycin, streptomycin, chloramphenicol, ampicillin, tetracycline, erythromycin, carbenicillin, imipenem, and spectinomycin [36,75,93,100,101]. The most effective antibiotic combination and its concentration are typically determined empirically for each specific case. It is desired that the antibiotic/antimycotic mix is well-tolerated by the isolated species and is efficient in inhibiting the growth of a broad range of bacteria. The effectiveness of antibacterial or antifungal treatments can be assessed by performing microbial analysis on nutrient agar supplemented with glucose and monitoring the number of bacterial colonies that grow despite the antimicrobial treatment [93,101]. Importantly, antibiotic treatment can affect photosynthetic activity and cell metabolism, as demonstrated in the filamentous cyanobacterium *Nostoc flagelliforme* [101].

Recently, a novel approach for microalgae culture purification using fluorescence-activated cell sorting (FACS) has been described. This method leverages the natural autofluorescence of chlorophyll-containing microorganisms [102,103]. By employing FACS, several axenic cultures of freshwater green microalgae and cyanobacteria were successfully obtained within approximately four weeks, significantly reducing the time required compared to conventional techniques [103].

In other instances, original samples from saline environments contain organisms that feed on microalgae, commonly referred to as ‘grazers’. These mainly unicellular organisms belong to protozoa (such as amoebas, flagellates, and ciliates), but also to multicellular animals, such as rotifers (although rare in saline environments) and crustaceans (e.g., copepods) [104,105,106]. In addition, recent research indicates that chironomid larvae disrupt microbial mats in the Sečovlje salterns by feeding on the cyanobacterium *Coleofasciculus chtonoplastes* [107]. However, macroscopic observation of these insect larvae allows for their removal during the initial stages of the isolation process. In hypersaline environments, the main grazers are the nanoflagellate *Halocafeteria seosinensis*, the ciliate *Fabrea salina*, and shrimps from the genus *Artemia* (e.g., *Artemia salina*) [10,108,109,110]. Since these organisms feed on microalgae, they prevent the culture from expanding, which poses a significant challenge in large-scale microalgae cultivation. Moreover, grazers can also rapidly destroy microalgae in environmental samples, complicating laboratory isolation and cultivation. For example, the euryhaline rotifer *Brachionus plicatilis* can consume up to 12,000 microalgal cells per hour [111].

Larger animals, such as copepods and rotifers, can be removed immediately after sampling using a plankton sieve or by gentle filtering [60,64]. In addition, rotifers are also sensitive to hydrodynamic cavitation [112]. Alternatively, microscopic zooplankton can be eliminated using different chemical compounds. For instance, *Dunaliella*-eating ciliates can be removed with quinine sulfate, which effectively targets the pests while minimally affecting *Dunaliella* health [113]. A combination of detergent and a foam flotation system has been successful in eradicating ciliates from (freshwater) *Chlorella* cultures [114]. Rotifers are sensitive to hypochlorite solutions and treatments involving Fe/H_2_O_2_ [115,116].

Since the number of microalgae that scientists have successfully grown in the laboratory is very low compared to their numbers in the natural environment, it is evident that many cyanobacteria and microalgae form obligate communities. In these cases, achieving axenic cultures of a single species or strain is practically impossible. For example, the marine nitrogen-fixing cyanobacterium Candidatus *Atelocyanobacterium thalassa* UCYN-A requires the presence of unicellular algae to survive as it lacks critical metabolic pathways that are complemented by the algae [117]. In marine environments, nitrogen-fixing cyanobacteria often form symbiotic relationships with various hosts, including microalgae (such as diatoms and haptophytes) and unicellular protists (such as dinoflagellates and ciliates) [118]. The existence of such mutual or parasitic interactions attenuates or even prevents the establishment of axenic cultures. Additionally, symbiosis affects different biosynthetic pathways [118]. Disrupting these relationships could alter the production of biologically interesting molecules in the culture. Therefore, the composition of mixed culture must be thoroughly investigated and described to avoid misinterpretation.

### 4.4. Axenicity Testing and Microalgae Identification

The purity of microalgal or cyanobacterial culture is typically monitored continuously throughout the entire isolation process to recognize the presence of contaminants and to confirm that the culture is axenic in later stages.

Various approaches can be used to assess culture purity during the purification process, including light microscopy, fluorescence microscopy or fluorescence-activated cell sorting (FACS) for detecting algal pigments, enriched broth tests, and DNA barcoding [64,119]. These methods differ in specificity and sensitivity. For instance, examining samples under a light microscope can quickly reveal microbial diversity and the presence of zooplankton, making it a useful technique in the early stages of isolation and during the purification process. Conversely, sequencing specific genomic regions, known as barcodes, is highly effective for species identification but can be challenging to apply to mixed cultures [119]. Additionally, monitoring culture health is crucial and can be achieved by determining algal growth. This can be conducted through cell counting under a light microscope or spectrophotometrically by measuring optical density [64].

When axenic cultures are obtained from environmental samples, the primary goal is usually to identify the isolated species. Historically, cyanobacterial identification has been based on morphological and phenotypic characteristics [62]. While this approach remains valuable today, it is often used in conjunction with modern techniques. Key morphological features to observe include (i) the presence of filaments, (ii) cell size and width, (iii) cell shape (common shapes are coccid, hemispherical, ovoid, rod-shaped, and cylindrical), (iv) the presence and arrangement of thylakoids, (v) cell aggregation (clusters of several cells), (vi) filament branching, (vii) the arrangement of cells within the filament, (viii) the presence of differentiated cells called heterocysts, (ix) the arrangement of filaments, and (x) the presence of extracellular sheaths [62]. Although microscopic observation alone can sometimes suffice, morphology-based identification is often insufficient. It is important to consider that cyanobacterial morphological features can change under laboratory conditions and may be influenced by cultivation settings and medium composition [61].

A powerful identification technique is DNA barcoding, which involves determining the nucleotide sequence of a specific genomic region and comparing it to reference sequences of known species [120,121]. For green microalgae, commonly used barcodes include the rbcL gene, 18S rDNA, and the internal transcribed spacer (ITS1 and ITS2) [100,122,123]. For cyanobacteria, identification is typically performed using the 16S rDNA gene or the 16S-23S rDNA ITS region [122,124]. For example, we could discriminate between 10 highly similar species/strains of *Synechocystis* members using DNA barcoding of the ITS/rDNA genomic segments [125].

## 5. Towards a Standardized Cultivation Pipeline for Saltern Microalgae

**Figure 3:**
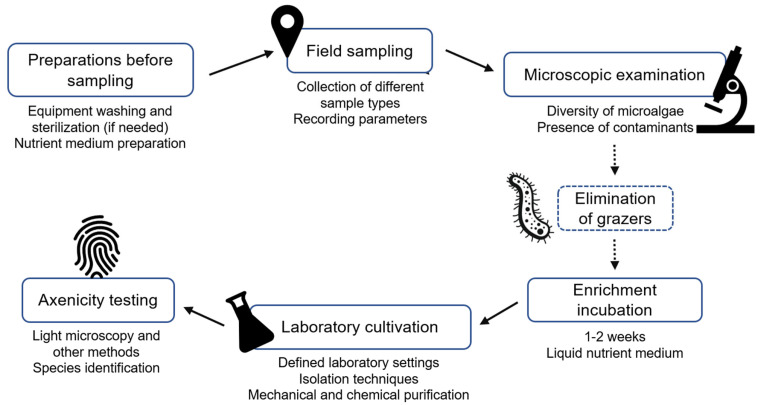
Workflow for the isolation and cultivation of microalgae and cyanobacteria from environmental samples, including those obtained in hypersaline environments. The workflow consists of: (1) pre-sampling preparation of sampling equipment, labware, and reagents; (2) field sampling to collect various sample types, including water, microbial mats, and crystallized salt; (3) microscopic examination of samples to assess diversity and detect contaminants; (4) elimination of contaminants, especially grazers (if no grazers are observed, proceed directly to Step 5); (5) enrichment of environmental samples under controlled conditions; (6) laboratory cultivation using appropriate isolation and purification techniques; and (7) verification of culture axenicity and species identification.

Preparing axenic cultures from environmental samples is often a difficult and time-consuming process with low success rates. The strategies used for cultivating and purifying microorganisms can limit the types and numbers of cyanobacteria and other photosynthetic microorganisms that can be grown as pure cultures in the laboratory. Another challenge is the lack of a standardized procedure for isolating hypersaline species, which could be systematically adopted by different research groups worldwide to increase the reliability of results and enable meaningful comparisons. To address this, we propose a workflow for isolating and cultivating microalgae from hypersaline environments, specifically from solar salterns, covering all steps from initial sampling to species identification in axenic culture (Figure 3). The proposed pipeline is based on the literature cited throughout this article and on our own work on microorganisms with biotechnological potential isolated from the Sečovlje Salt Pans.

### Steps of the Proposed Workflow

Preparations Before Sampling

- Wash sampling instruments and storage containers;
- Sterilize all utensils and glassware for laboratory work (dissecting tools, handling instruments, growth flasks, etc.) to ensure aseptic conditions;
- Prepare selected nutrient media and sterilize them by autoclaving or filtering.

Field Sampling

- Record sampling parameters, including location coordinates, water temperature, pH, and salinity;
- Collect different sample types: water from crystallization pools, microbial mats (if present), and crystallized salt samples (if present and *Dunaliella* is expected);
- Ensure quick transfer of samples to the laboratory to maintain sample integrity.

Post-Sampling, Pre-Cultivation

- Examine crude environmental samples under a stereomicroscope to inspect the structure of solid and semi-solid samples and the eventual presence of macroscopic grazers, followed by light microscopy to assess the diversity of microalgae and cyanobacteria and to detect contaminants, such as heterotrophic organisms and zooplankton;
- If grazers are observed, promptly apply suitable purification techniques, such as filtering or chemical treatments, to remove them. This step is essential and must be completed before cultivation as the success of the subsequent isolation depends on the removal of these organisms;
- Define the laboratory setup (growth medium composition, temperature, illumination, agitation settings, etc.) based on the sampling parameters and microscopic observations. It is recommended to test multiple conditions in parallel to facilitate the isolation of various strains from the same field sample.

Laboratory Cultivation

- Apply stringent axenic practices throughout the entire isolation and cultivation process to prevent contamination. This includes the use of sterilized equipment, sterile handling methods, and working in a clean environment;
- Enrich crude environmental samples by a 1–2-week incubation in selected liquid nutrient media under the chosen laboratory conditions. For petola mat samples, use only the upper green layer, which contains the photosynthetic microorganisms, to avoid contamination with microorganisms present in the lower layers. During the enrichment stage, avoid high light intensities to prevent culture damage;
- Regularly observe enriched environmental samples under a light microscope, with particular attention to the presence of filamentous cyanobacteria, which may require atypical growth conditions (see next step);
- Use appropriate isolation techniques to obtain a specific microorganism (e.g., a desired species present in the sample). The choice of technique should be empirically determined based on sample and target specifics. If multiple morphologically distinct species are observed, consider using different approaches in parallel to isolate all of them effectively. Most strains can be isolated using the streak plate method or by single-cell isolation with micropipetting. For filamentous cyanobacteria, consider using a cultivation method involving non-nutrient agarose plates overlaid with a liquid nutrient medium;
- Apply various purification techniques to achieve a unialgal or unibacterial culture. Select between mechanical and chemical methods, or use a combination of both, depending on the specific requirements of the culture;
- Gradually adjust the salinity of the nutrient medium. For samples containing filamentous cyanobacteria, start with a medium of lower salinity to prevent the salinity-induced inhibition of oxygenic photosynthesis. For *Dunaliella* species, begin with an intermediate salinity medium and slowly increase the salinity to promote the growth of more halophilic strains;
- Environmental samples from salterns, particularly petola, are likely to be highly biodiverse, consisting of various cyanobacteria and microalgae coexisting in equilibrium. Under laboratory conditions, however, the fastest-growing species tend to dominate and outcompete others. Therefore, it is advisable to maintain enriched samples over extended periods (up to six months) and to perform serial isolations of the predominant species at different time points as the composition of the mixed culture may change in response to the physiological requirements of different species.

Culture Monitoring

- Regularly check the purity of cultures. This can be routinely conducted by light microscopy. However, adapt the method of purity assessment according to the specific conditions and capabilities of your laboratory;
- Track microalgal growth by measuring optical density spectrophotometrically or by performing microscope-assisted cell counting;
- Once unialgal or unibacterial cultures are confirmed through light microscopy, apply more sensitive methods to verify culture axenicity. If possible, perform DNA barcoding for accurate identification of the isolated species.

## 6. Conclusions

Obtaining axenic cultures of photosynthetic microorganisms from environmental samples can be challenging, particularly due to the lack of standardized and well-established protocols in the existing literature. With this review, we provide simplified, step-by-step instructions for isolating green microalgae and cyanobacteria from hypersaline environments, with a focus on salterns. Our primary goal is to equip both new and experienced researchers in the field of halophilic microorganisms with essential procedures for isolation and laboratory cultivation, offering practical tips and empirical insights from our own laboratory experience.

However, we emphasize the importance of adapting protocols to the specific characteristics of the sample in question. The identification of a particular microalgal or cyanobacterial species in culture should guide the subsequent steps. Additionally, we encourage researchers to explore various isolation techniques and cultivation parameters in parallel to achieve the best results.

In summary, the proposed pipeline for isolating and cultivating microorganisms from hypersaline environments holds several promising applications:

I. In biotechnology, the isolated microorganisms can be harnessed for their unique enzymes and metabolites, which are often more stable and active under extreme conditions. These enzymes and bioactive compounds can be applied in industries such as pharmaceuticals, biofuels, and agriculture;
II. In fundamental research, our pipeline provides researchers with the tools to study the adaptive mechanisms of extremophiles at a molecular level. Understanding how these microorganisms survive and thrive in high-salinity environments can offer insights into fundamental biological processes and evolutionary adaptations;
III. For industrial applications, photosynthetic microorganisms isolated from hypersaline environments can be exploited in processes that require stability under harsh conditions, such as high salt concentrations. Their application can enhance the efficiency and sustainability of production processes;
IV. In biodiversity and conservation, by isolating and cultivating these microorganisms, researchers can contribute to the documentation and preservation of microbial diversity in hypersaline environments. This knowledge can inform conservation strategies and help protect these unique ecosystems.

The pipeline’s flexibility and adaptability make it a valuable tool for researchers with varying levels of experience, enabling them to explore a wide range of applications and advance our understanding of life in extreme environments.

## Figures and Tables

**Table 2:** Overview of the main isolation techniques that can be applied to microorganisms from hypersaline environments, along with their advantages and disadvantages.

Isolation Technique	Description	Advantages	Disadvantages
Serial dilution	Diluting the original sample in a series of dilution steps then plating the diluted sample to obtain individual colonies.	Cost-effective, simple.	Time-consuming, requires multiple rounds to achieve purity.
Streak plate	Inoculating solid medium by streaking with a loop a small amount of the sample in a zig-zag pattern.	Easy to perform, cost-effective, visual selection of colonies.	Not suitable for all types of cyanobacteria and microalgae, requires a range of specific media.
Filtration	Passing the sample through a filter to separate microorganisms based on size.	Straightforward, suitable for larger volumes.	Requires a set of sieves with specific pore sizes for different microorganisms. Often results in the clogging of sieves.
Flow cytometry	Separating cells based on their fluorescence (and/or other) properties using a dedicated apparatus.	Highly accurate, high throughput.	Expensive equipment, requires technical expertise.
Antibiotic treatment	Adding antibiotics to a growth medium to selectively inhibit the growth of undesired microorganisms.	Useful for removing bacteria.	Target microorganisms have to be resistant.
Density gradient centrifugation	Using a gradient of sucrose to separate microorganisms based on their buoyant density during centrifugation.	Separates types of microorganisms with different densities.	Requires specialized equipment, can be time-consuming.

## Data Availability

The original contributions presented in this study are included in the article. Further inquiries can be directed to the corresponding author.
